# The development and psychometric evaluation of vaccine hesitancy and perception scale towards COVID-19 vaccination (VHAPS-CV19)

**DOI:** 10.3389/fpubh.2026.1730659

**Published:** 2026-02-18

**Authors:** Amanda L. Meiklejohn, Mintesnot T. Teni, Megan Kalinowski, Michael Poirier, Divya S. Subramaniam

**Affiliations:** 1 Department of Health and Clinical Outcomes Research, Saint Louis University School of Medicine, Saint Louis, MO, United States; 2 Department of Epidemiology and Biostatistics, Saint Louis University College for Public Health and Social Justice, Saint Louis, MO, United States

**Keywords:** coronavirus, COVID-19, instrument development, vaccine hesitancy, vaccine uptake

## Abstract

The purpose of this study is to develop a COVID-19 vaccine hesitancy measurement instrument. Literature demonstrated a theme of seven constructs to include in the instrument: benefits, cost, confidence, convenience, complacency, risk, and perception. Face and content validity was performed using subject matter experts, self-identified COVID-19 hesitant, and non-hesitant individuals. The pilot survey data were collected from the end of December 2022 to May 2023 via an online convenient sample (*n* = 352). Test re-test analysis was performed 2 weeks following the initial survey (*n* = 73). After the test re-test and factor analysis, 29 items among two factors emerged. Factor 1 (vaccine hesitancy) includes three sub-domains (confidence, complacency, and convenience). Factor 2 (perception and trust) includes five sub-domains (perceived benefit, barrier, susceptibility, severity, and trust). This study illustrates the development of the vaccine hesitancy scale may assist policymakers and healthcare providers in the reasoning behind vaccine hesitancy towards COVID-19 vaccines. This research adds an instrument to assist public health practitioners measure COVID-19 vaccine and perception among their patient population.

## Introduction

Vaccination is one of the most effective public health interventions in history, preventing millions of deaths annually from infectious diseases such as measles, influenza, and pertussis through direct protection and community immunity. Vaccines are estimated to avert 3.5–5 million premature deaths each year worldwide, yet confidence in vaccines varies across populations and contexts, contributing to suboptimal uptake for both routine and novel vaccines such as those developed for COVID-19 (1).

Despite the overwhelming evidence demonstrating vaccine safety and effectiveness, many individuals continue to perceive vaccines as unsafe, unnecessary, or mistrust them due to misinformation and sociocultural factors (2). Vaccine hesitancy (VH), or “the delay in acceptance or refusal of vaccination despite the availability of vaccination services” has become a growing public health concern with WHO including it as one of the top 10 threats to global health (3, 4).

The COVID-19 pandemic, caused by the SARS-CoV-2 virus first detected in late 2019, has led to substantial morbidity and mortality worldwide and continues to pose public health challenges. Severe outcomes from COVID-19 include hospitalization, long-term health sequelae, and death; vaccines developed since 2020 have significantly reduced these risks and remain an important strategy to mitigate disease burden (5). However, uptake of updated COVID-19 vaccines, including annual booster formulations, has been low in the United States and other high-income settings. As of April 2025, approximately 20.4% of U.S. adults reported receiving the 2024–25 COVID-19 vaccine, with substantial variability by age and perceived disease risk (6). This decline in uptake can be partly attributed to the reduced effectiveness of the vaccines and vaccine hesitancy due to misinformation (7–9). Overall, these trends highlight persistent barriers related to vaccine confidence, perceived necessity, and evolving guidelines.

Several sociopolitical developments in 2025 have further complicated public perceptions of vaccines. In the U.S., regulatory adjustments narrowed recommended access to COVID-19 vaccines for select populations (i.e., adults aged ≥65 years and individuals with underlying conditions), and some advisory committees revised guidance on indication and prioritization, contributing to confusion among the public and clinicians (10). Beyond traditional measures of hesitancy, COVID-19–related stress, anxiety, and psychological distress have also been linked with vaccine attitudes in several studies. Instruments such as the COVID Stress Scale (CSS) and related derivatives have been employed to capture emotional and cognitive determinants of hesitancy in addition to measures focused on beliefs about safety and efficacy. These broader constructs reflect that attitudes to vaccination are embedded in broader psychosocial contexts that may influence decision-making (11, 12).

Given the evolving nature of COVID-19 vaccination recommendations, the ongoing threat of vaccine-preventable diseases, and the persistent prevalence of vaccine hesitancy, there is a critical need for robust measurement instruments that capture contemporary attitudes, beliefs, and perceptions related to COVID-19 vaccination. This study aimed to develop and psychometrically evaluate the Vaccine Hesitancy and Perception Scale toward COVID-19 Vaccination (VHAPS-CV19) to support public health research and intervention strategies in the current phase of the pandemic.

## Methods

### Conceptual framework

VH is a multifaceted concept, encompassing different behavioral categories such as confidence (referring to trust in the vaccine or healthcare provider), complacency (perceiving the need for vaccination or valuing it), and convenience (relating to accessibility) (13). A variety of conceptual frameworks have been used to develop measurements for vaccine hesitancy in previous studies. The widely used models include the Health Belief Model (HBM), the Theory of Planned Behavior (TPB), and the Trust, Confidence, and Cooperation (TCC) Model (2).

For our instrument, we adopted a hybrid conceptual framework that integrates the core dimensions of VH (confidence, complacency, and convenience), the HBM, and the TCC model. We decided to use the HBM as vaccine hesitancy is primarily affected by an individual’s perceived beliefs, including perceived benefit, perceived susceptibility, perceived severity, and perceived barriers (14, 15). These perceptions are closely tied to trust in vaccines, an essential determinant of VH (3, 14, 15). The TCC model was incorporated to capture the role of trust more fully in promoting cooperation and vaccine acceptance (14–16).

This integrated conceptual framework, with two main constructs, Vaccine Hesitancy and Perceptions of Vaccines, guided both our literature search strategy and the identification of relevant VH measurement items. The Vaccine Hesitancy construct includes three subdomains (confidence, complacency, and convenience), while Perceptions of Vaccines encompasses five subdomains (perceived benefit, perceived barrier, perceived susceptibility, perceived severity, and trust) (14–16).

### Development of the instrument

The purpose of this study was to develop an instrument to be used to measure vaccine hesitancy of COVID-19 vaccines. Health professionals and other responsible groups can use the information to distribute and administer the COVID-19 vaccines. Furthermore, this will benefit the development of health education programs toward vaccine hesitancy, specifically for COVID-19 vaccines. To date, there is no gold standard COVID-19 vaccine hesitancy scale. Hundreds of scales have been developed to identify key factors of vaccine hesitancy for COVID-19 (8). The gold standard vaccine hesitancy scale (VHS) was created in 2012 by the WHO SAGE Working Group, answered by parents about vaccination practices for their children (17). The questions were developed from several global pilot tests and literature reviews of similar instruments, including the Parent Attitude About Childhood Vaccines (PACV). This scale design allows for the deployment of the scale in diverse groups and cultures (18). However, a general vaccine hesitancy scale based on the perceptions of parents regarding vaccinating their children is not sufficient to address the unique hesitancies surrounding the COVID-19 vaccine. With its rapid development and distribution, the global nature of the pandemic, and its politicization, a COVID-19 vaccine hesitancy scale that captures the unique circumstances of COVID-19 has yet to be developed.

After the comprehensive literature review guided by the conceptual framework, the research team combed through hundreds of pertinent questions and statements related to vaccine hesitancy. Literature for COVID-19 vaccine hesitancy demonstrated a theme of seven constructs: benefits, cost, confidence, convenience, complacency, risk, and perception (3, 8, 9, 19). The items were developed based on previous scales, including the WHO-SAGE vaccine hesitancy measurement, generalized vaccine hesitancy scale and HPV vaccination attitudes and beliefs scale, and other relevant literature (2–4, 8, 9, 19). Using common themes found in the literature, the research team narrowed down the number of questions to 71 questions deemed vital and relevant. After the items were generated, duplicate items and items that were deemed irrelevant were removed.

### Content validity index

To validate the VHAPS-CV19, a content validity analysis was conducted in partnership with subject matter experts (SMEs). This phase’s primary focus is on determining the content validity of this survey tool. The items in this survey were based on literature collected during the initial phase of the study. Each item was clearly written and determined to be representative of the hesitancies and perceptions of interest identified by the research team. Feedback from SMEs would inform the inclusion, revision, and exclusion of items from the finalized tool.

SMEs were instructed to rate the relevance of each item directly in a Google Sheet, ranging from not relevant to highly relevant. 10 SMEs were recruited and asked to review the content validity of the survey tool. SMEs across the United States were chosen based on their demonstrated expertise and mastery related to COVID-19 and associated vaccination. The SMEs include professors, clinicians, and public health professionals, with experience ranging from 2 years to more than 10 years. Demographic information was not collected as part of this process.

The initial VHPSC-19 contained 41 items and was developed to identify factors related to COVID-19 vaccine hesitancy. The items were further reviewed by subject matter experts in the content validity index (CVI) phase using a Likert scale rating. The following formulas were used to calculate results for I-CVI and S-CVI scores (20):

- I-CVI = N of experts rating items a 3 or 4 / Total N of experts
- S-CVI = Final average of all I-CVI scores

### Face validity

To test whether each item was measuring what the research intended, face validity was also performed using self-identified COVID-19 hesitant and non-hesitant individuals. The laypersons who were asked to provide feedback on the items were previously known to the research team. Individuals provided feedback about the wording, structure, and content of the survey. The research team considered the feedback for the final items.

### Pilot testing: sample and recruitment

For the pilot testing, American adults aged 18 years of age or older living in the United States were eligible to complete the VHPSC-19 via a Qualtrics online survey. Participants’ recruitment was conducted through Social Media pages (which will include Twitter, Facebook, Instagram, LinkedIn) and through institutional listservs. The surveys were completed anonymously. Upon completion, participants were asked voluntarily to provide name and email address for the raffle purpose. There was no way to link survey responses to the name/email provided. Once the winners were selected, all names and emails were permanently destroyed. Participation in this study was voluntary. All respondents gave their online consent before completing the survey. Data were collected from the end of December 2022 to May 1, 2023. Ethical approval was obtained from the institutional review board of our institution. The total number of respondents was 352. Due to the convenience sample and snowballing recruitment method, we are unable to confirm an accurate response rate. Participants were also invited to participate in a test–retest survey 2 weeks following their initial survey submission to assess the test–retest reliability of the tool. An alpha value of over 0.7 was used to determine which items to retain (21), 73 respondents fully completed the test–retest survey 2 weeks after the initial survey. Following reliability analysis testing with the alpha values over 0.7, 29 items were retained. Six items were dropped, which were repetitive or did not meet the alpha value cutoff of 0.7 (21).

### Data analysis

All responses were used for analysis after management procedures were applied for missing data. The authors used SPSS, version 28.0, software (SPSS Inc.) to perform descriptive analysis of demographic characteristics and EFA and internal consistency and scale reliability testing. The researchers utilized principal axis factoring, a prevalent estimation approach in exploratory factor analysis (EFA), to offer an elucidation for the underlying composition among the items (22). The researchers employed the varimax rotation technique, which is widely recognized in exploratory factor analysis (EFA), with the underlying assumption that there was no correlation among the items. This choice was made to enhance the presentation of the factors inherent in the VHPSC-19, thereby contributing to a more meaningful representation (23).

## Results

### Content validity

The scale level (S-CVI) was 0.87. The item level (I-CVI) ranged from 0.71 to 1.00. The CVI process led to the retention of 35 highly relevant items. The remaining 35 items were tested with a survey that asked a diverse sample of adult Americans living in the United States to specify their level of agreement or disagreement on a Likert scale of 1 to 5 (*strongly disagree* to *strongly agree*) with statements that led to assess a person’s hesitancy towards the COVID-19 vaccine. Demographic questions were included.

### Demographic characteristics

352 participants completed the survey. Most of the participants were Female (71.6%), Married (42.6%), White (79.5%), had at least a bachelor’s degree (78.0%) and working full-time (62.2%). Their ages ranged from 24 to 76 years (*M* = 46.14 years; SD = 12.02 years). Half (49.7%) aligned with democratic political views. Ninety-three percent of participants had at least one COVID-19 vaccine, and 86.9% received a COVID-19 vaccine booster. Almost two-thirds of the participants reported they received positive COVID-19 test results (Table 1).

**Table 1:** Demographics of participants in the pilot survey (*N* = 352).

Variables	Total (*N* = 352)
Age	
24 and younger	82 (23.3%)
25–34	143 (40.6%)
35–44	44 (12.5%)
45 and older	83 (23.6%)
Race	
White or Caucasian	279 (79.5%)
Asian	34 (9.7%)
Black or African American	18 (5.1%)
Other	20 (5.7%)
Gender	
Female	252 (71.6%)
Male	89 (25.3%)
Other	11 (3.1%)
Education level	
Bachelor’s degree	142 (40.6%)
Graduate Degree	131 (37.4%)
Less than college degree	77 (22.0%)
Political alignment	
Democrat	175 (49.7%)
Republican	59 (16.8%)
Independent	56 (15.9%)
Other	62 (17.6%)
Religion	
Agnostic	60 (17.0%)
Atheist	34 (9.7%)
Christian Scientist	14 (4.0%)
Jewish	12 (3.4%)
Other	75 (21.3%)
Protestant	77 (21.9%)
Roman Catholic	80 (22.7%)
Marital status	
Divorced/Separated/widowed	27 (7.7%)
Living with a partner	43 (12.2%)
Married	150 (42.6%)
Never been married	132 (37.5%)
Employment status	
Working part-time	24 (6.8%)
Student	93 (26.4%)
Working full-time	219 (62.2%)
Other	16 (4.5%)
Place residence	
Major metropolitan area	115 (32.7%)
Medium-sized city	127 (36.1%)
Rural	52 (14.8%)
Small-sized city	58 (16.5%)
Household income	
$150,000 or more	81 (23.0%)
$100,000–$149,999	53 (15.1%)
$75,000–$99,999	45 (12.8%)
$50,000–$74,999	59 (16.8%)
$25,000–$49,999	42 (11.9%)
Less than $25,000	39 (11.1%)
Prefer not to say	33 (9.4%)
Have you had a COVID-19 vaccine	
No	24 (6.8%)
Yes	328 (93.2%)
Have you received a COVID-19 booster?	
No	43 (13.1%)
Yes	285 (86.9%)
Have you ever received a positive COVID-19 test result?	
No	122 (34.7%)
Yes	230 (65.3%)

### Extraction, interpretation, naming, and scoring of factors

An EFA with varimax rotation was performed to determine the correlations and underlying structure among the factors. All 352 participants were included in the EFA of the 29 items within the VHPSC-19. All factors with eigenvalues of 0.30 or greater were retained and further evaluated. Pearson correlation coefficients for the items ranged from 0.980 to 0.986 (*p* < 0.001). The Kaiser-Meyer-Olkin Measure of Sampling Adequacy was 0.961 and Bartlett’s Test of Sphericity was significant at x^2^_(406, 352)_ = 8,025.78, *p* < 0.001, validating that factor analysis was suitable for this study sample (23). Following the EFA with varimax rotation, a three-factor solution emerged and explained 62.3% of the total variance. The loadings in the factor component matrix were between −0.831 and 0.881, while the loadings in the factor rotated component matrix were between 0.351 and 0.838; both indicated minimal to excellent loading (24). While three factors emerged, Factor 3 did not consist of a minimum of three unique statements. Items 16 and 26 were dropped from the tool as they only appeared in Factor 3. This rationale supported the choice to maintain the two-factor solution. Items with double or triple loadings were placed in the most appropriate factor, given their importance to understanding factors and existing theoretical foundations. Next, Cronbach’s alpha and item-total correlation were used to measure reliability and internal consistency. For the 14 items from factor 1, the internal consistency value ranged from 0.948 to 0.962, and the alpha value was 0.955. For the 12 items from factor 2, the internal consistency value ranged from 0.917 to 0.939 and the alpha value was 0.929, indicating excellent reliability (25) (see Table 2).

**Table 2:** Factor pattern of principal axis factoring method: varimax with Kaiser normalization rotation for VHPSC-19 items.

Item No.	Items	Factor 1: COVID-19 vaccine perception and trust	Factor 2: COVID-19 vaccine hesitancy	Item–total correlation
6	It is important to get the COVID-19 vaccine to prevent coronavirus spreading in the community.	0.838	–	0.875
2	Getting a COVID-19 vaccine is a good means to protect myself from the COVID-19 disease.	0.811	–	0.838
20	I would get the COVID-19 vaccine as soon as possible if it was offered multiple convenient locations (e.g., grocery store, church, school, pharmacy, doctor office) near me.	0.801	–	0.832
1	Taking a COVID-19 vaccination is important for my overall health and well-being.	0.797	–	0.837
3	If I get the COVID-19 vaccine, it will help to protect my family and friends against the coronavirus.	0.794	–	0.831
4	COVID-19 vaccine will stop the spread of coronavirus.	0.788	–	0.752
21	I would get the COVID-19 vaccine as soon as possible if it was offered to me at work.	0.761	–	0.772
12	I trust scientists to give me reliable information on the benefits and risks of the COVID-19 vaccine.	0.715	–	0.786
7	I am willing to pay for the COVID-19 vaccine (i.e., out of pocket cost).	0.672	–	0.689
5	The COVID-19 vaccine will reduce the severity of symptoms if I get the coronavirus.	0.671	–	0.745
11	I trust healthcare providers and health professionals to give me reliable information on the benefits and risks of the COVID-19 vaccine.	0.664	–	0.736
9	Generally, I do what my doctor or healthcare provider recommends about COVID-19 vaccines.	0.656	–	0.654
13	I trust vaccine manufacturers to give me reliable information on the benefits and risks of the COVID-19 vaccine.	0.639	–	0.668
10	I trust the Government to give me reliable information on the benefits and risks of the COVID-19 vaccine.	0.610	–	0.642
18	I am concerned about a COVID-19 vaccine causing severe adverse reactions (e.g., severe allergic reaction, death, etc.).	–	0.754	0.698
8	I am worried about getting vaccinated because I already had COVID-19.	–	0.582	0.673
24	I am afraid of getting the COVID-19 vaccine.	–	0.731	0.661
25	Getting the COVID-19 vaccine makes me feel anxious.	–	0.734	0.641
29	I refuse COVID-19 vaccination because my family member or guardian is against the COVID-19 vaccine.	–	0.594	0.641
15	The COVID-19 vaccine safety data is untrustworthy.	–	0.705	0.522
14	The COVID-19 vaccine is promoted mainly because of manufacturers’ profit.	–	0.718	0.485
23	I refuse to get the COVID-19 vaccine.	-	0.632	0.455
17	If a person has already had COVID-19, they do not need to get a vaccine.	–	0.772	0.450
28	I do not consider vaccination against COVID-19 an effective way to deal with the COVID-19 pandemic.	–	0.732	0.377
22	Vaccination against coronavirus is not required, because I am confident that my immune system will cope with this infection.	–	0.675	0.351
19	I am concerned about one of any of the long-term side effects of getting a COVID-19 vaccine (e.g., infertility, thyroid dysfunction, change in my DNA, myocarditis, etc.)	–	0.631	0.302

After finalizing the VHPSC-19, the authors reviewed and named the two factors. Scores are determined by summing up the ratings of each item on the 5-point Likert scale (where 1 = lowest, 2 = low, 3 = middle, 4 = high, and 5 = highest). Factor 1 included 14 items regarding COVID-19 vaccine perception and trust, which are interconnected. Poor perception and lack of trust of a vaccine can be due to misinformation, poor health literacy, mistrust in government officials, and improper health communication for the given audience. The score of Factor 1 ranged from 14 (lowest concerns for vaccine perception and trust) to 70 (highest concerns for vaccine perception and trust). Factor 2 included 12 items related to COVID-19 vaccine hesitancy. This factor measures a person’s reluctance or hesitation towards getting the COVID-19 vaccine due to cost, complacency, confidence, and convenience. (Table 3) The score of Factor 2 ranged from 12 (lowest vaccine hesitancy) to 60 (highest vaccine hesitancy).

**Table 3:** Internal consistency (Cronbach’s Alpha), mean, standard deviation, and reliability index (standard deviation of each factor) for VHSPC-19.

Factor	Number of Items	Cronbach’s Alpha	*M*	SD	Variance
1	14	0.955	54.48	12.946	167.597
2	12	0.929	23.18	9.828	96.582

## Discussion

Vaccines are considered one of the greatest public health achievements of the last century. In the last century alone, it is estimated that vaccines have saved 2–3 million lives each year (1). Recent surveys have identified an elevated level of hesitancy for the COVID-19 vaccine (26–28). One survey conducted during the Coronavirus pandemic revealed that approximately 3 in 10 adults were unsure if they would accept the vaccine and 1 in 10 did not intend to be vaccinated against COVID-19 (27). The VHPSC-19 scale was created to help public health officials identify barriers to vaccination uptake. Due to the novelty of COVID-19, a gold standard scale does not currently exist, and it will take time to evaluate which scales effectively and appropriately measure vaccine hesitancy for COVID-19 (26).

Developing a COVID-19 vaccine hesitancy scale can help unify existing vaccine hesitancy frameworks by providing a disease-specific tool to identify population groups experiencing COVID-19–related vaccine hesitancy and to inform targeted interventions, such as health education, aimed at addressing negative perceptions of vaccination. The development of this survey tool is motivated by the continued incidence of death because of COVID-19. As time continues to pass since the onset of the pandemic, public perception of the importance of being vaccinated can change and persist (28, 29). Understanding what factors influence COVID-19 vaccine hesitancy is a crucial step towards increasing vaccination uptake rates for the COVID-19 vaccine. COVID-19 vaccine hesitancy is a unique and new type of vaccine hesitancy, so it is important to accurately measure COVID-19 VH appropriately. Two factors were identified in this model to assess COVID-19 vaccine perception, trust, and hesitancy.

While COVID-19 vaccine perception and trust are intricately linked to COVID-19 vaccine hesitancy, there are distinct differences. Factor 1 (vaccine hesitancy) includes three sub-domains (confidence, complacency, and convenience). For Factor 1, making vaccines more accessible, engaging with healthcare professionals, providing clear and accessible information, transparency, incentives, and rewards will all aid in reducing rates of COVID-19 vaccine hesitancy.

Factor 2 (perception and trust) includes five sub-domains (perceived benefit, barrier, susceptibility, severity, and trust). For Factor 2, COVID-19 perception refers to how an individual (or community) perceives the COVID-19 vaccine and understands the safety, efficacy, and importance of protecting the spread of COVID-19 in their community. COVID-19 vaccine trust refers to an individual’s trust in the government or healthcare systems that are supporting or providing COVID-19 vaccines, which may also include pharmaceutical companies and regulatory authorities that develop and approve vaccines. To overcome a person who has low COVID-19 vaccine trust and negative perceptions of the COVID-19 vaccine, the health community must provide accurate and clear information about COVID-19 vaccines, engage with the community and healthcare providers to build trust, provide personalized recommendations, address peer and community norms, and combat misinformation.

Past vaccine hesitancy instruments have focused on “the three Cs,” which have previously been able to measure an individual’s vaccine hesitancy (30). During the COVID-19 pandemic, a new factor of trust and perception for the COVID-19 vaccine emerged. This was also observed in our scale development. Current literature consists of scales measuring COVID-19 vaccine hesitancy, a majority of which only included one or two of “the three Cs” of vaccine hesitancy. Not only did we include items to capture these components, but we also included another factor that emerged during the COVID-19 pandemic, which is trust and perception.

The next phase of this study will include further testing and validation of the VHPSC-19 scale using a general population study sample. The VHPSC-19 could help identify key components leading to vaccine hesitancy among the population.

## Limitations

Demographic data was not collected for the SMEs that provided feedback. The results provided by the SMEs may not be generalizable to all SMEs in the field. Another limitation was the method of SME feedback. Using Google Sheets, responses were not anonymous, which allowed other SMEs to see responses from their peers. We utilized a convenience sample, which limited the demographics that were included. This is an online questionnaire, so we are unable to determine how accurately people are answering the questions and whether the sample is representative of the general population. In addition, we did not take into consideration the timeline of historic events and current hesitancy for the COVID-19 vaccine. We would expect that back in March 2020, the vaccine opinion of the public would differ compared to December 2020. In addition, it would have been interesting to have questionnaire data around a new vaccine pre-COVID. Future studies should further investigate other determinants that could influence an individual’s vaccination uptake. For example, a questionnaire asking the public if they would take a new vaccine if a new virus naturally appeared in the population. We also would like to include questions about people who follow the CDC guidelines and are less hesitant than those who do not. For example, we would predict a person who is practicing social distancing and wearing a mask appropriately would be less hesitant for a COVID-19 vaccine compared to someone who does not practice social distancing and does not wear a mask appropriately. There is an indication vaccine hesitancy will continue to be an issue for years to come (31).

### Implication of practice and conclusion

As literature indicates, there is no gold standard to measure COVID-19 vaccine hesitancy and perception currently in the United States. To overcome COVID-19 vaccine hesitancy, public health practitioners first need to be able to identify those who are hesitant or have negative perception. This instrument assist public health professionals in their ability to identify a person’s COVID-19 vaccine hesitancy status as well as COVID-19 vaccine perception. Based on how a person responds, a different intervention may need to be used to overcome COVID-19 vaccine hesitancy.

## Data Availability

The raw data supporting the conclusions of this article will be made available by the authors, without undue reservation.
